# Cure of Crooked Nose by Subcutaneous Division of the Cartilages

**Published:** 1843-03

**Authors:** 


*Cure of Crooked Nose by Subcutaneous Division of the Cartilages.*—By Prof. Dieffenbach.—[Dr. Dieffenbach remarks that the wrynose is either a natural deformity, or is caused by accident. He has operated in two cases with complete success: in one the deformity was congenital, and in the other it was caused by a fall. He thus describes the operation:]

With a small curved bistoury I made a puncture by the side of the bridge of the nose, at the point of union between the cartilage and the bone, the bistoury was then carried under the skin, so as to separate the cartilage of the side and bridge of the nose from the bone. By a second puncture, on the other side, the middle partition of the nose and the cartilage of that side were divided.

The nostrils were then stuffed with lint, and the nose retained in its proper position with strips of plaster. The parts healed quickly, without inflammation or suppuration.

*N. Y. Lancet* from *Gaz. Med.*

